# Development of the Better Research Interactions for Every Family (BRIEF) intervention to support recruitment for neonatal clinical trials: an intervention mapping guided approach

**DOI:** 10.1186/s13063-024-08446-6

**Published:** 2024-09-12

**Authors:** Elliott Mark Weiss, Megan M. Gray, Linda K. Ko, Devan M. Duenas, Ellie Oslin, Stephanie A. Kraft

**Affiliations:** 1 Treuman Katz Center for Pediatric Bioethics and Palliative Care, Seattle Children’s Research Institute, 4800 Sand Point Way NE, M/S FA.2.113 Neonatology, Seattle, WA 98105 USA; 2 Department of Pediatrics, University of Washington School of Medicine, Seattle, WA USA; 3 Department of Health Systems and Population Health, University of Washington, Seattle, WA USA; 4 Department of Bioethics and Decision Sciences, Geisinger College of Health Sciences, Danville, PA USA

**Keywords:** Neonatal clinical trials, Recruitment, Research ethics, Intervention

## Abstract

**Background:**

Recruitment for neonatal clinical trials can be particularly challenging. Low enrollment rates bias the research population and decrease generalizability of findings. We identified a critical need for an intervention to improve how researchers recruit for neonatal clinical trials. Working within the US neonatal research context, we developed the Better Research Interactions for Every Family (BRIEF) Intervention, which had two overarching goals: to improve the recruitment experience for all parents, focusing on minoritized populations, and to increase participation, focusing on decreasing disparities in research participation.

**Methods:**

We used intervention mapping (IM) to guide all steps of intervention development. IM is a planning framework that provides a systematic process and detailed protocol for step-by-step decision-making for intervention development, implementation, and evaluation.

**Results:**

We performed IM’s six steps. In step 1, we convened two stakeholder groups, a parent panel and an expert panel, who provided guidance through development of all BRIEF components. Through a recent systematic review, empirical data collected by our team, and consultations with the panels, we identified key determinants (barriers and facilitators) of low enrollment rates and research team members as change agents. In step 2, we iteratively refined our list of key factors to include and linked determinants of behavior changes to these performance objectives. In step 3, we chose three theories (social cognitive theory, theory of information processing, and the trans-theoretical model), methods from identified practical applications suitable for the population (research team members) and the context (busy research NICU teams). In step 4, we developed and refined the intervention components, including self-guided pre-work and a single in-person session. In step 5, we identified the Darbepoetin plus slow-release intravenous iron trial as our partner study in which to pilot BRIEF. In step 6, we developed a multi-stage evaluation plan that included five distinct levels of outcomes.

**Conclusions:**

This manuscript shares our rationale and processes for the creation of a research team member-facing intervention aiming to improve recruitment processes for neonatal clinical trials. Our approach can inform those aiming to improve recruitment for neonatal clinical trials and those who may be considering use of IM within similar contexts.

**Supplementary Information:**

The online version contains supplementary material available at 10.1186/s13063-024-08446-6.

## Background

Challenges enrolling participants are common across all types of clinical trials [1, 2]. In the neonatal intensive care unit (NICU), recruitment challenges are compounded by high clinical acuity, inclusion of a particularly vulnerable population, and short enrollment windows during a time when the parents are likely experiencing high levels of stress [3–5].

Low enrollment for neonatal clinical trials is problematic because it is not random: those who enroll differ clinically from those who do not. Sicker infants may be less likely to participate than less sick infants [6]. Furthermore, although the data are limited, those available suggest demographic disparities in neonatal research with lower participation of Black infants [7–10]. These disparities are particularly concerning because of known worse health outcomes for minoritized racial and ethnic groups in US NICUs [11–14].

In recruitment for neonatal clinical trials, research team members must identify potentially eligible infants, approach their parents, and carry out all components of consent [15]. Through the processes and interactions that are part of the recruitment for neonatal clinical trials, parents decide whether to enroll their infant. This decision is multifactorial, influenced by factors including the following: barriers and facilitators of participation that affect parents’ conception of reasons for and against enrollment [16], parents’ experience of being approached for research [17], and parents’ perceptions of the trustworthiness of the research and clinical teams [18, 19] which may be informed by their experience of the respectfulness of research teams [20, 21]. Thus, optimal recruitment for neonatal clinical trials requires that research teams use a recruitment process that addresses the many factors that shape parents’ decisions.

We, therefore, identified a critical need to develop an intervention focused on improving the recruitment process for neonatal clinical trials. We had two overarching goals. First is to improve the recruitment experience for all parents, focusing on minoritized populations [22]. Second is to increase participation, focusing on decreasing disparities in research participation.

In this paper, we describe our process for developing the Better Research Interactions for Every Family (BRIEF) Intervention and its evaluation plan, within the US neonatal research context. This paper can inform regulators, funders, and clinical trialists on how to build a novel research team member-facing educational intervention to improve research recruitment practices for NICU research.

## Methods

We used intervention mapping (IM) to guide all steps of the development of our intervention. IM [23] is a rigorous technique for integrating theory and empirical findings to develop health interventions. It provides a systematic process and detailed protocol for effective, step-by-step decision-making for intervention development, implementation, and evaluation [24]. IM ensures that the intervention matches the priority population’s social and environmental needs. Several components of an intervention can be bundled to address multiple levels of change. Application of theories is essential to link population needs to behavior change methods to operationalize the methods into practical steps for implementation. Thus, IM allows for the creation of multi-component interventions that may be evaluated for effectiveness of the overall bundle as well as each component. IM consists of six steps: (1) needs assessment, (2) creation of matrices of behavioral determinants and performance objectives, (3) choosing theoretical methods and practical strategies, (4) designing the intervention, (5) creation of an adoption and implementation plan, (6) creation of an evaluation plan.

IM has been used to build interventions to decrease disparities in research participation in other populations [25–27]. For example, the Randomized Recruitment Intervention Trial (RECRUIT) was an intervention aimed at increasing the inclusion of minoritized populations within clinical trials conducted in specialty clinics. The RECRUIT intervention was created using IM methods [25] and tested via cluster-randomized design across 50 specialty sites [26]. RECRUIT findings were equivocal, with the primary outcome not significant but a suggestion of improvement in recruitment of people from minoritized populations for 3 out of 4 studied trials [28]. IM was also used for the creation of an intervention for HIV clinical trial participation [27]. To our knowledge, IM has not previously been used in the NICU population.

## Results

### Step 1: Needs assessment

We started the needs assessment process by creating two relevant stakeholder groups to advise intervention development, literature review, and data collected by our team. One of these groups was our expert panel, which consisted of individuals across the US with expertise in bioethics, neonatal clinical trials, stakeholder engagement, adult education, health disparities, qualitative methods, and intervention development. The other group was our parent panel of 10 former NICU parents from the Pacific Northwest Seattle region; more than half were from minoritized populations and more than half had experience being recruited for neonatal research. Parent panel members were paid for their participation. These two groups provided guidance through all steps of the project’s development.

The needs assessment consisted of a literature review supported by a recent systematic review of barriers and facilitators to participation in pediatric research [16] (see Table 1) and a review of findings from previous work conducted by our team members, including surveys [8, 9] and interviews of parents approached for NICU research [17, 19], interviews [21] and Delphi surveys [29] around issues of respect in research, and interviews with pediatric research coordinators [15, 22].

**Table 1:** Literature review supporting development of BRIEF intervention

	Performance objectives	Supporting literature
Pre-approach	PO.1. Partnership with clinical team	Parental desire for greater involvement of clinical team [8, 17, 30–33] Ethical argument of increased partnership between research and clinical teams [34, 35]
	PO.2. Partnership with bedside nursing	Key role that nurses play in supporting parents in the NICU [36, 37]
Initial connection	PO.3. Family names	Asking family preferred names, pronouns, nicknames [15]
	PO.4. Options for discussing research	Importance of initial interaction with research team [38] Stress of having discussion at NICU bedside [39]
	PO.5. Empathy with the NICU family experience	Empathy from research team as important [32]
	PO.6. Family needs	Feeling overwhelmed at time of approach [40–42] Unaddressed stress and anxiety at time of approach as reason to decline [43–46] Attention to family needs [47]
Building connection	PO.7. Research team’s investment in trial	Reason team is doing study as key information [48]
	PO.8. Benefit for future infants	Specific to others with their child’s illness [49, 50] Past research as beneficial to current care [38] Participation as helping future infants [46, 51, 52]
	PO.9. Options for participation	Supporting enrollment decision-making during times of stress [53, 54] Risks and benefits clearly described [55] Potential burdens to family described [56]
Follow-up	PO.10. Ongoing connection with family	Value of ongoing parent engagement with research [57, 58] Important for family to know who to contact for questions Research participants often want continued engagement after initial research approaches [21]

We chose to structure the BRIEF intervention to follow our previously developed 4-stage conceptual framework of factors impacting relationship building in the researcher-participant-surrogate recruitment interaction within pediatric research [15, 22]. That framework developed out of interviews with pediatric research team members guided by a model of trust-building within community academic research partnerships [59]. Our prior conceptual work identified the relational level (i.e., interactions between research team members and parents) as central to relationship-building between pediatric research team members and parents of potential participants. Our framework delineated four temporal stages of relationship building: pre-approach (deciding whether, when, and how to approach a family), initial connection (starting the interaction with parents), building connection (ongoing interaction, including discussing the study), and follow-up (closing the interaction, preparing for next steps, and working towards positive longer-term relationships) [14]. For BRIEF, we prioritized considerations of equity (present at all levels of the framework) [15, 22], in order to support increased inclusion of and improved experience for minoritized and marginalized populations. We did this by designing our contributing studies, in particular our interviews of parents approached for neonatal research (#2 in the following paragraph), so that minoritized and marginalized populations were over-sampled to assure their views were included.

We were informed by four recent empirical studies performed by our research teams. These include (1) surveys of parents approached for a single neonatal clinical trial, (2) interviews of parents approached for several neonatal research studies, (3) interviews of adult participants in genomic research, and (4) a Delphi survey process with patients in community clinics. We will briefly describe these four lines of inquiry and their contribution to the development of BRIEF.

First, we were informed by recent surveys of parents who were asked to participate in a neonatal clinical trial [8, 9]. We had conducted surveys at 12 US NICUs with parents of infants who enrolled in the *H*igh-dose *E*rythropoietin for *A*sphyxia and encepha*L*opathy (HEAL) trial [60] or who were eligible but declined enrollment. In these surveys, most parents, but particularly those who chose not to participate in HEAL, wanted greater involvement of the clinical team when learning about research. Important items included the central role of a positive relationship with members of the research team, altruism as a major motivator for participation, and challenges around the right timing for approaching a family for a neonatal clinical trial.

Second, we recently completed a project interviewing parents approached for neonatal research, including both those who participated in and those who declined participation in neonatal research. Key findings from this work included parents’ desire for greater involvement of the clinical team in learning about research and the essential role of emotions and relationships in enrollment decision-making [17]. Motivations for participation included altruism and a hope for benefit to their infant; reasons against participation included burdens of participation to the family and perceived risk to infants [19].

Third, we were informed by recent interviews with participants in genomics research about respect [21]. Findings revealed domains of respect that illustrate the importance of attending to how researchers demonstrate respect across the entire research process. Specific key findings included connecting with families, showing empathy during interpersonal interactions, and responding to the family’s needs. Additionally, presenting the option to participate or not in a neutral way was seen as a way researchers may illustrate respect.

Finally, we were guided by findings from a modified Delphi survey with patients in community clinics about priorities for feeling respected in research [29]. These asked respondents to identify the most important factors for demonstrating respect in this setting. The highest ranked were as follows: receiving information to make a decision and positive interpersonal interactions, reiterating the importance of improving the recruitment and consent process, including the interpersonal relationships that take place during this process.

**Fig. 1:**
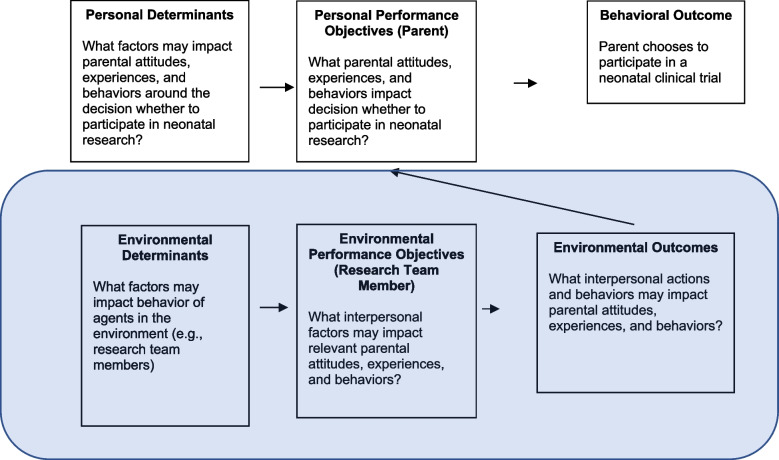
Logic model of change: targeting “environmental level” of research team members. For BRIEF, our team decided to focus on the environmental level (e.g., directly impacting behavior of research team members) rather than personal level (e.g., directly impacting behavior of parents)

The IM framework differentiates between the “personal level” at which an individual performs a particular behavioral outcome and the “environmental level,” the social and physical environment where the individual exists. Applying the IM framework to the current project, the research team members exist at the “environmental level” and serve to impact change through their social interactions and providing a welcoming physical space to parents (see Fig. 1). Parents then are the individuals at the “personal level” making the decision that leads to the behavioral outcome of interest: whether or not they choose to participate in neonatal research. Importantly, our team agreed that the parent recruitment experience was vital and valuable independent of their ultimate enrollment decision. While an increased enrollment rate is an important goal to increase the representativeness of the research population, each family must be free to make the enrollment decision best aligned with their values. Therefore, we determined that we must measure outcomes related to the parental recruitment experience.

Informed by these data as well as a literature review on the topic, we created a logic model of change for the development of the BRIEF intervention (see Fig. 1). With our expert panel, parent advisory panel, and partner clinical trialists, we set out to determine the appropriate agent of change for our intervention. We identified that the intervention could directly target: (a) parents deciding whether to participate in neonatal research, (b) research team members, or (c) both. We decided to focus the BRIEF intervention on research team members. This choice reflects the importance of the relationship developed between researchers and potential parents approached for consent, including the researchers’ responsibility to improve parents’ experience and address disparities in research participation.

For neonatal clinical trials in the US, there is wide variability by site regarding who approaches families for participation [61]. This may include the following: the physician primary investigator, physician co-investigator, research coordinator, research assistant, and research nurse. These individuals may have anywhere from extensive to no knowledge about clinical medicine, may or may not be part of the infant’s medical team, and may or may not have deep knowledge of the research. To be broadly applicable across sites, we designed BRIEF to be useful for all individuals approaching families for neonatal research regardless of their role, training, and experience.

### Step 2: Creation of matrices of behavioral determinants and performance objectives

We drew on the previous qualitative research done with research participants and research staff (as described above) to identify key features of respectful, equitable recruitment interactions. We conceptualized our environmental outcomes within our previously created framework, in which we delineated four stages of relationship building in pediatric clinical research (pre-approach, initial connection, building connection, and follow-up) [14]. The identified key features represent the environmental outcomes we hoped to improve with our intervention.

The integration of determinants from step 1 led to several major findings that align with the constructs of the social cognitive theory (SCT). First, parents wanted to learn about research including participants’ risks, benefits, and research burden through a closer interaction with the research team (behavioral capability and outcome expectations). Second, parents believed that arming themselves with information about research would increase their confidence to make an informed decision (self-efficacy). Third, parents needed to be emotionally ready to enroll in research and valued emotional empathy from the research team (emotional coping responses, expectancies, determinism). Fourth, parents were motivated to participate in research when they understood the benefits for their infants and/or were guided by altruism (behavioral capability and outcome expectations).

SCT proposes that human actions and behaviors are influenced by the dynamic interplay between three groups of determinants: personal, behavioral, and environmental factors [62]. This theory focuses on the interaction between internal personal factors such as attention, memory, and motivation and external factors such as the opportunity for a desired outcome. A key component of SCT is self-efficacy, defined as an individual’s belief that they are capable of performing a behavior [63]. Behavior change therefore is determined by the interaction and alignment between outcome expectations (e.g., an individual’s belief that their behavior will result in a particular outcome) and self-efficacy expectations (e.g., an individual’s belief that they can produce that outcome). Research team members responsible for obtaining consent for neonatal clinical trials are highly motivated to improve their ability to connect with families and meet them at the stage of the families’ readiness for research but often feel limited in their skills to create a positive environment for parents. We selected SCT because its constructs align with the environmental needs of parents deciding to enroll in neonatal trials, and by addressing the determinants, we will increase the capacity and skills of the research team to create an environment that is conducive to positive research recruitment for parents.

**Fig. 2:**
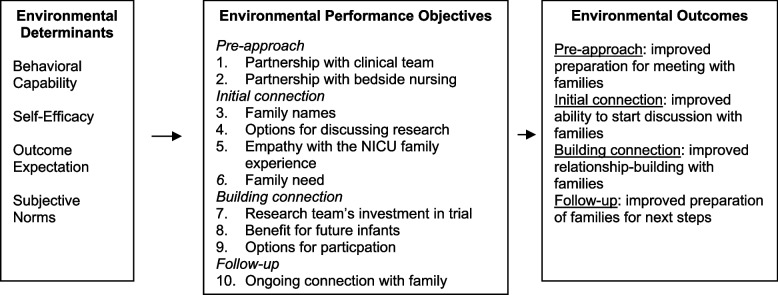
Logic model of change: selection of environmental determinants, performance objectives, and outcomes

For BRIEF, we selected four SCT determinants of behavior change: behavior capability (knowledge and skills to perform task), self-efficacy, outcome expectation, and subjective norms. These were prioritized based on relevancy to the problem and changeability with the potential intervention. We then began identifying and prioritizing performance objectives through multiple rounds of discussions and meetings with our expert panel and our parent advisory panel. We met with each group separately multiple times over a 9-month period to present our initial list of 16 items and then received feedback both in the group meeting and through email correspondence with individual members. Over this period, the items were iteratively refined to a final set of 10 performance objectives with extensive input from our parent and expert advisory groups. Figure 2 shows the selected outcomes, performance objectives, and determinants.

We next began linking determinants to performance objectives (see Table 2). For example, performance objective 2 asks the research team member to meet with the bedside nurse before approaching the family and use that interaction with nursing to support the early steps of relationship-building. We identified four determinants to support research team members doing this effectively: demonstrate the ability to identify and engage with the bedside nurse and share information gained with the family (behavioral capability), express confidence in learning about the patient and family situation from nursing and verbalize knowledge gained to family (self-efficacy), expect that nursing may have important insights into how the family would prefer to be approached about research (outcome expectation), and expect that nursing will respond positively to engagement with the research team (subjective norms).

**Table 2:** Environmental outcome matrix

Environmental outcome: improvement in…	Performance objective: research team member…	Determinants			
		Behavioral capability (knowledge and skills)	Self-efficacy (belief in capability)	Outcome expectation	Subjective norms
Pre-approach	PO.1. Demonstrates to the family that the study team is partnered with the patient’s clinical team	BC.1. Demonstrates ability to identify and contact appropriate treating clinician	SE.1. Expresses confidence in ability to meet with clinician and explain this connection to the family	OE.1. Expects that families may be more open to hearing about research if they feel their clinical team is supportive	SN.1. Clinicians will respond positively to request to inform family about research
	PO.2. Builds on relationship between family and bedside nursing to support introductory relationship-building	BC.2. Demonstrates ability to identify and engage with bedside nurse and share information gained with family	SE.2. Expresses confidence in learning about the patient and family situation from nursing, and in verbalizing knowledge gained to family	OE.2. Expect that nursing may have important insights into how family would prefer to be approached about research	SN.2. Nursing will respond positively to engagement with research team
Initial connection	PO.3. Initiates relationship with infant and caregivers by learning how they would like to be addressed and addressing them accordingly	BC.3. Describes how to obtain information about preferences names from medical chart, confirms with family and uses correct terms, pronunciations, and pronouns	SE.3. Expresses confidence in learning how the patients and family prefer to be addressed	OE.3. Expects that confirming and using correct names, pronunciations, and pronouns will help families feel respected	SN.3. Families will respond positively to being asked about how they prefer to be addressed
	PO.4. Provides families choices about when and where research discussion occurs	BC.4. Individualizes discussion of options by anticipating family’s needs, seeking preferences, and following through to meet preferences	SE.4. Expresses confidence in individualizing conversation surrounding different options for participation	OE.4. Expects that families may be more open to hearing about research when given control over when and where discussion occurs	SN.4. Families will respond positively to being asked preferences around research discussion
	PO.5. Acknowledges that the NICU is a challenging place for families and support relationship-building through empathizing with family experiences	BC.5. Anticipates and explores family NICU experience, recognizes and responds to subtle emotional cues, and provides empathic support	SE.5. Expresses confidence they can ask about and respond sensitively to the family NICU experience	OE.5. Expects that families may feel more connected to a research team member who expresses empathy for what they are going through	SN.5. Families will respond positively to being asked about NICU experience
	PO.6. Supports relationship building by identifying, exploring, acknowledging, and addressing family needs	BC.6. Describes how to identifies needs through preparation and directly asking family needs; demonstrates ability to respond to emergent needs	SE.6. Expresses confidence that they can ask parents needs and can identify appropriate resources	OE.6. Expects that families may be more fully able to consider research after other needs are addressed	SN.6. Families will respond positively to being asked their needs
Building connection	PO.7. Effectively communicates why this research team is invested in this particular research	BC.7. Formulates a compelling message on how this trial fits within the larger goals of the research team	SE.7. Expresses confidence in explaining why the research team believes the study goals are important	OE.7. Expects that learning research team passion and motivation may encourage families to participate	SN.7. Families will respond positively to knowing research team motivations
	PO.8. Shares the rationale for research including how it may benefit future infants	BC.8. Describes how research may benefit future NICU infants; connects to family’s goals and hopes	SE.8. Expresses confidence in explaining goals of this research	OE.8. Expects that families may be more interested in research if understand future benefits to other	SN.8. Families will respond positively learning that research may help future infants
	PO.9. Demonstrates that the decision whether to participate is the family’s choice and that families make different choices	BC.9. Individualizes discussion by eliciting family’s values and experience to support their decision to participate or not participate	SE.9. Expresses confidence in having conversations about family’s decision to participate or not and support decision	OE.9. Expects that providing reasons for and against participating may help families feel the research team respects their choice	SN.9. Families will respond positively learning that whatever choice they make will be supported
Follow-up	PO.10. Provides the information families need to make it easy and comfortable for them to follow-up with the research team	BC.10. Describes contact information for follow-up; knows how to refer if questions require responses from others	SE.10. Expresses confidence in sharing contact info and bringing in others when necessary	OE.10. Expects that providing contact information and referring to others when appropriate will help families feel supported	SN.10. Families will feel more supported if ongoing contact is genuinely offered and straightforward

### Step 3: Choosing theoretical methods and practical strategies

After identifying SCT-based determinants, the research team moved to step 3 in which we selected theory-informed methods and practical applications through which the determinants may be influenced. We chose methods from three theories: SCT, theory of information processing [64, 65], and the trans-theoretical model [66]. These theories were chosen largely based on our review of prior literature of similar interventions to improve recruitment processes in other settings [25–27] as well as input from our expert panel members with experience and expertise in IM and in intervention development.

As the behavioral determinants captured personal as well as environmental factors, we were guided by the construct of SCT to help identify the performance objectives that align under each of the four stages of relationship building.

We chose to inform the development of the BRIEF materials using the information processing theory as the research teams play the role of information receivers (receiving information on how to best present neonatal research to families) and givers (giving information to families in an effective manner). This enabled our team to think about how information from BRIEF would be processed by the research team to help research teams anticipate how research information will be processed by the families. The theory of information processing suggest that individuals do not simply respond to stimuli but process information based on structural features of the incoming information [64, 65]. The act of processing information is impacted by such things as where the information is stored, cognitive processes to transfer stored information, and awareness of how information is processed [67, 68]. We chose this theory to optimize our presentation of critical information as part of the pre-work (e.g., chunking key items into a four step model) how we formatted our synchronous session (e.g., guided discussion to support learning of material), as well as provide opportunities for role play to practice these skills.

The findings from step 1 showed that families have different amounts and aspects of information about research as well as emotional readiness to engage in research. Research teams approaching these families need to be aware of their stage of readiness for research participation and provide information that will meet the needs of families to motivate behavior change. The trans-theoretical model suggests that behavior change is a conscious and intentional process that occurs over six stages: precontemplation, contemplation, preparation, action, maintenance, and termination [66]. Progress through stages occurs through constructs of motivational readiness, processes of change, self-efficacy, and decisional balance [69]. These constructs describe why and how a behavior change occurs and can be utilized to support desired behaviors [70, 71]. From this theory, we were able to create a process whereby we could identify and respond to participants’ varied stages of readiness to change (e.g., from pre-work responses) in order to support the synchronous session.

We then identified practical applications suitable for the population (research team members) and the context (busy NICU research teams). Table 3 shows the methods chosen and relevant practical applications, which in turn supported the components of the intervention. Components to support these were chosen: pre-work including didactics, personal stories from parents, example scenarios, and self-reflection and a single synchronous session including didactics, sharing of personal experiences, and role-play practice scenarios. All components were directly linked to one of the ten specific performance objectives and a location within our conceptual framework.

**Table 3:** Theoretical methods and practical applications

SCT determinants	Theoretical methods	Practical applications
Behavioral capability (knowledge and skills)	Skills training^a^	Synchronous meeting didactics Group work: mapping recruitment processes Practice scenarios using standardized actors
	Persuasive communication^a^	Video pre-work Synchronous meeting
	Individualization^b^;	Tailoring didactics to respondents’ pre-work Tailoring case to target partner neonatal clinical trial (DIVI)
	Stages of readiness to change^b^	Flexibility of live session to respond to respondents’ shared experiences and beliefs
	Chunking^c^	Use of visuals for mapping of recruitment processes and POs Grouping of performance objectives into four stages
	Discussion^c^	Eliciting experience, attitudes, and beliefs during live session
Self-efficacy (individual’s belief about ability to perform task)	Modeling^a^	Video pre-work: examples of using POs Synchronous practice scenarios: watching others use POs
	Active learning^a^	Synchronous meeting sharing experiences, challenges, successes
	Implementation intentions^a^	Making if–then plans for complicated situations
Outcome expectation	Modeling^a^	As above
	Active learning^a^	As above
	Elaboration^c^	Synchronous meeting sharing experiences, challenges, successes
Subjective norms	Modeling^a^	As above

^a^Social cognitive theory

^b^Trans-theoretical model

^c^Theory of information processing

### Step 4: Designing the intervention

We developed and refined the intervention components, including self-guided pre-work and a single in-person synchronous session. Educational videos were developed in collaboration with the Seattle Children’s Marketing and Communications team. Videos included NICU parents and research coordinators sharing their experiences and perspectives and scripted skits depicting hypothetical research recruitment approaches between research coordinators and NICU parents. The pre-work also included self-reflection questions to increase engagement and prepare learners for the in-person session. Thinkific, a software platform for the creation and delivery of online courses [72], was used to develop the pre-work where participants would engage with the intervention materials by learning about the performance objectives, viewing educational videos, and answering a series of questions inquiring about their experience with recruitment and the performance objectives. Because the target audience includes anyone on a research team that would recruit participants (i.e., investigators and coordinators), we aimed to use a platform for the pre-work that would maximize accessibility (e.g., allow for access at any time and from any location). The online pre-work platform was completed within three weeks prior to the in-person session.

Each research team member participated in a single 2-h group in-person session. This format was selected to maximize the efficacy of the role-play exercise, optimize engagement, and support team building and was felt to be feasible with a single-site neonatal clinical trial. The intervention occurred in a small group format (target 4 or 6 participants per session) to enable 2 or 3 partner break-out groups as well as ample opportunity for discussion in the larger group. Sessions occurred in a conference room at the medical center with audiovisual capabilities. Components were created to be easily adaptable to a remote synchronous setting (e.g., videoconferencing) which would likely be needed for expansion to other settings. Our team developed four role-play scenarios (two scenarios, each with two distinct characters being approached for a different neonatal research study). Each of the four scenarios was created to facilitate practice with a sub-set of the performance objectives (see Fig. S1). They were designed around two neonatal clinical trials to allow the practice to apply across different trials.

The synchronous session was divided into four sections: introduction, an overview of the conceptual model, and didactics; team member discussion of reflection questions from pre-work; role plays; and closing discussion with a feedback survey. The intervention was designed to be piloted in the NICU, but applicable to multiple recruitment settings, and to achieve two goals: (1) improve the recruitment experience for families and (2) increase enrollment rates (and decrease disparities in enrollment). All components were created iteratively in collaboration with our parent panel and expert panel members. The synchronous session was led by the project lead and lead author of the current manuscript.

Improving the research experience for parents from marginalized and minoritized populations was a central goal of this project. Because of this, as we have described, we prioritized information from these groups in the identification of our performance objectives and the development of our content. However, we must recognize that parents from marginalized and minoritized populations are highly heterogenous with variability in whether and which additional challenges they face. Because of this, we did not recommend researchers change their approach solely based on parental demographics. Our team and our parent and expert advisors felt such broad generalizations would be inappropriate.

### Step 5: Creation of an adoption and implementation plan

Next, we identified a partner neonatal clinical trial to pilot the BRIEF intervention. For partnership, we identified the single site Darbepoetin plus slow-release intravenous iron (DIVI) [73]. DIVI is a phase II trial to demonstrate Darbepoetin’s feasibility, safety, and potential benefit with different iron formulations and its impact on transfusion requirements and on neurodevelopmental outcomes for infants born before 32 weeks gestational age. Logistical and methodological advantages included broad inclusion criteria, the need for early enrollment (within the first three days of life), and a team highly committed to supporting this pilot-phase collaboration, which was essential to get feedback to be used for the creation of a version to be used across a multi-site clinical trial.

In future iterations, we will test the BRIEF intervention at multiple sites and for multiple neonatal clinical trials. To maximize the applicability across settings, we will endeavor to include studies with variety across key components of neonatal research, including the following: inclusion criteria, enrollment window (including whether prenatal consent is possible), intervention characteristics, and follow-up duration. When this is done, we will need to create an adoption and implementation plan specific to the settings chosen.

### Step 6: Creation of an evaluation plan

To evaluate the intervention, we developed a multi-stage evaluation plan that included five distinct levels of outcomes.

Data at the infant level (P1) included the dichotomous outcome of whether the infant ultimately participated in the DIVI study. We also designed a targeted data extraction for all infants eligible for the DIVI study at birth in order to collect demographic and medical information to evaluate for any differences between DIVI participants and DIVI non-participants. Selected demographics include race, ethnicity, insurance status, gestational age, birthweight, and sex. Selected medical information include key prenatal factors (maternal prenatal antibiotics, prenatal steroids), key factors at birth (Apgar scores, resuscitation needed), and disposition to discharge.

Two outcomes were specific to the parent experience level. We invited all parents who were approached by the DIVI team for a recruitment discussion to participate in a one-time REDCap-based parent survey (P2). The rationale of this outcome was to evaluate parents’ experience of being recruited to participate in the DIVI study with a focus on areas addressed by the BRIEF intervention, to gather demographic information, and to inquire about parents’ willingness to participate in a follow-up interview. As part of the IM process, the survey development was centered on assessing parental experience around each of the ten performance objectives present in the BRIEF intervention through an iterative process including input from our parent advisory panel and expert panel as well as cognitive interviews with NICU parents to assess interpretation of our questions. We developed two novel 5-point Likert scale questions for each performance objective (total 20 questions), in addition to novel questions about related higher level constructs (e.g., respect, trustworthiness, equity), a validated scale for trust in medical researchers [74], and respondent demographics.

Parents who participated in our survey were invited to participate in in-depth parent interviews (P3). The rationale of this outcome was to evaluate the parent experience of being recruited to participate in the DIVI study with a focus on areas addressed by the BRIEF intervention and to establish qualitative data on the parent experience. The interview guide was also structured around the ten performance objectives and related constructs with a similar process as for the survey questions described above.

The next outcome level focused on the interaction between parents and research team members. For a subset of parents approached, we gathered data from the parent-research team member recruitment discussions (P4). The data included two components: audio-recordings of the recruitment discussions between the DIVI team members and the parents and research team member self-assessment of the sample sub-set of DIVI recruitment discussions using the BRIEF Assessment Tool (Fig. S2), which was created as part of the IM process. The BRIEF Assessment Tool included 10 Likert scale questions, each linked to a specific performance objective. The rationale for these outcomes was twofold: first was to compare DIVI team members’ self-assessment of their achievement of each performance objective before and after BRIEF intervention training, and second was to compare DIVI team members’ self-assessment with external assessment of audio-recorded recruitment discussions using the same tool.

Our final outcomes were specific to the research team member level (P5). These included a multi-phase data collection process consisting of surveys after the pre-work, surveys after the synchronous session, and interviews at the project’s close. The rationale of these outcomes was to evaluate the DIVI team members’ experience of the BRIEF intervention so we may refine it for future iterations.

As of the writing of the current manuscript, data collection for all five levels of outcomes for this single-center pilot assessment of BRIEF within the partner DIVI trial has been completed. Analysis is ongoing, and our findings are currently being drafted, after which they will be submitted as manuscripts to be considered within peer review medical journals.

## Discussion

This paper describes the planning process of the BRIEF intervention using IM. This rigorous process allowed us to develop an intervention based on the best available evidence and to monitor outcomes as they map on to each of our performance objectives.

While there is interest in improving recruitment for neonatal trials, we were unable to identify published accounts of rigorously developed and/or evaluated interventions with this goal. Creating, evaluating, and implementing such interventions is critical to moving the field forward. This project represents the first recruitment intervention for neonatal clinical trials utilizing IM.

Our decision to target research team members was a strength of BRIEF for several reasons. First, there is a strong ethical justification for researchers to take action to improve research diversity and inclusion. Second, these individuals are highly motivated and reachable, given other training they must do, such as onboarding for a neonatal clinical trial. Third, there is currently huge variability in role, training, experience, and approach to recruitment for these studies, suggesting potential for improvement with standardization.

Our creation and use of multi-level outcomes will determine which performance objectives were most effective in order to refine the intervention for future use. After completing the BRIEF intervention within DIVI, we hope to refine the intervention based on our pilot results and test it within a multi-site neonatal clinical trial.

We also hope to expand the BRIEF intervention into other contexts. Much of the conceptual and theoretical framework may be used within other settings, but we will assess for context-specific additional performance objectives and any additions that may be needed to our conceptual model. Potential disciplines for expansion of BRIEF include other settings where decisions need to be made quickly in a high-stress situation, such as emergency medicine and both adult and pediatric intensive care. BRIEF may also have value in other settings where trustworthiness and relationship building are especially important, such as within genomics and pain research.

### Limitations

While the development of the intervention prioritized empirical and conceptual work centering marginalized and minoritized populations, the intervention itself was created to broadly support all of those approached for neonatal research. Future work may consider if specific modifications to recruitment processes should be considered to best support certain populations. This project was developed within the context of US neonatal research. Applicability outside the US may vary, due to differences in recruitment approaches for neonatal research as well as differences in the social construction of marginalization in other contexts.

## Conclusion

The BRIEF intervention is a rigorously developed research team member-facing intervention aiming to improve recruitment processes for neonatal clinical trials. In describing our rationale and process for the creation of BRIEF using IM, we offer a guide for those considering using this approach to improve recruitment for neonatal clinical trials and other research settings as well as for those who may be considering use of IM in other contexts.

## Supplementary Information

Supplementary Material 1: Fig. S1. Scenarios for BRIEF in-person session.

Supplementary Material 2: Fig. S2. BRIEF Assessment Tool.

Supplementary Material 3.

## Data Availability

Deidentified data can be available on written request to Dr. Weiss. Depending on the nature of request, it may require approval by the UW IRB.

## Abbreviations

BRIEF: Better Research Interactions for Every Family
DIVI: Darbepoetin plus slow-release intravenous iron
HEAL: *H*Igh-dose *E*rythropoietin for *A*sphyxia and encepha*L*opathy
IM: Intervention mapping
NICU: Neonatal intensive care unit
RECRUIT: Randomized Recruitment Intervention Trial
SCT: Social cognitive theory
