# Supplementary material for: Development of the Better Research Interactions for Every Family (BRIEF) intervention to support recruitment for neonatal clinical trials: an intervention mapping guided approach
Source: Trials. 2024 Sep 12;25:610. doi: 10.1186/s13063-024-08446-6 (PMC11395641; doi:10.1186/s13063-024-08446-6)
Supplement: Supplementary file 1 — Supplementary Material 1: Fig. S1. Scenarios for BRIEF in-person session. [file 13063_2024_8446_MOESM1_ESM.docx]

Figure 3. Scenarios for BRIEF in-person session [online Supplement]

**Scenario 1: demonstrating partnership with clinical team**

***target POs: Partnership with clinical team [1], partnership with bedside nursing [2], family names [3]***

***Research team member instructions***

As we shared, we’ve learned that many families appreciate when the study team is partnering well with the clinical team—specifically their infant’s treating physician and bedside nurse.

In this scenario, you are a research team member approaching [Jessica][Kenzo], whose 26-week newborn, Jack, now 10 hours old, is eligible for the DIVI study. You’ve had time to check in with attending Dr. Valdez, who thinks this family will likely be open to research participation, and with bedside nurse Sam, who told you [mom][dad] is at bedside now.

For this scenario, we ask to prioritize sharing with [Jessica][Kenzo] the work you’ve done before entering the room: contact you’ve been able to make with the medical team and nurse and learning baby Jack’s and parents Jessica and Kenzo’s names.

***Standardized parent script***

Mom Jessica had a difficult pregnancy with lots of complications. This is her first living child after multiple miscarriages. She is very anxious about her baby, Jack, who was born urgently at 26 weeks in setting of placental insufficiency. Her husband, Kenzo, is in the military and just arrived back home today and came directly to the hospital. Jessica trusts Dr. Valdez, whom she met three weeks ago during a prenatal visit for threatened premature delivery, which Kenzo joined by phone. Jessica feels well supported by Sam, Jack’s nurse, but does wish that Sam didn’t have another sick baby to be taking care of as well. She has a hard time being called “mom” because it reminds her of a prior loss, so she appreciates being called by her first name. Jessica has shared with Kenzo her trust in the medical team and her anxieties around the pregnancy and birth. Kenzo is grateful that Jessica has such a good medical team and is feeling very protective of her. He also shares her anxieties about Jack’s health.

[Jessica][Kenzo] starts off closed off, giving only short answers. As the team shares a deeper connection with Dr. Valdez and Sam, [s]he warms up and engages more with the researcher. [S]he might ask “oh, what did they tell you?” in a curious but somewhat skeptical way if the researcher doesn’t specify what they learned from talking with their care team. If the researcher effectively illustrates that they understand the connection Jessica has with members of Jack’s medical team, [s]he might respond by warming up and sharing how important they have been in Jack’s care and supporting Jessica through it.

For Jessica: If the researcher gives several options for how to refer to Jessica, she might disclose “I appreciate you asking; I actually don’t like being called ‘mom.’ Jessica works!” If the researcher starts by calling her mom without checking what her name is, she might begin to disengage, look away, and give curt answers.

For Kenzo: If the researcher give several options for how to refer to Kenzo, he might disclose “I appreciate you asking; I’m fine with dad or Kenzo, but my wife would prefer not to be called ‘mom’ so first names are probably best.” If the researcher starts by calling him dad without checking, he might begin to feel distrustful, disengage, and give curt answers.

***Facilitated group discussion***

Were you able to bring up recent discussion with Dr. Valdez and Sam? How did it feel? What went well? What other ways could this be done? Other feedback on this?

**Scenario 2: initial connection to family**

***target POs: options for discussing research [4], empathy with NICU experience [5], family needs [6]***

***Research team member instructions***

We’ve also learned that families often want to feel that the research team is respectful of the stressors of being a parent in the NICU.

We will continue the discussion with [Jessica][Kenzo] whose 26-week newborn, Jack, now 10 hours old, is eligible for the DIVI study. You’ve shared that Dr. Valdez and Sam are supportive of you approaching the family and they seem open to learning more about the DIVI study. In your short chat with Sam, Sam mentioned that they were anxiously awaiting results from an ultrasound performed an hour ago.

For this scenario, we ask you to prioritize empathizing with the NICU family experience and exploring family needs as you provide options for when and how to discuss the research study.

***Standardized parent script***

*See* ***scenario 1*** *for background about Jessica and Kenzo.*

Jessica and Kenzo have been waiting for results of an ultrasound that their baby got earlier that day. Jessica has been reading online about what the different results could mean for her baby’s brain development, and Kenzo is trying to convey a sense of calm to her but underneath he is also scared. As you start this scenario, please quickly pivot the conversation to your anxiety around this test and whether the researcher might know what the ultrasound results are, or when they will be back.

If the researcher jumps right into the conversation about research without acknowledging the clinical setting and the family’s concerns, [Jessica][Kenzo] might respond angrily, with statements like “I really can’t talk about that right now” or “No thanks, not interested,” similar to how [s]he might respond to a telemarketer call. As the researcher makes empathetic statements and shows they are actively listening, [s]he might transition into sharing how scared [s]he is about the ultrasound and in general. If the researcher acknowledges how hard it is to wait for results of the ultrasound, [s]he might open up more and say “It’s just so hard to think about anything else while I’m waiting. They said they would be back later today but I don’t know what ‘later’ means.” As the researcher shows more and more empathy, [s]he might say “I really appreciate you taking the time to listen to me. I know this isn’t your job.” If they specifically offer to check in with the clinical team about the ultrasound, [s]he might say “That would be so helpful, thank you. I know you said you were coming to talk about a research study for Jack and I’m sorry I’m so distracted, but I do want to hear about it once I get the results.”

***Facilitated group discussion***

How did it feel to have a parent ask you a clinical question? How did responding feel? What went well? What other ways could this be done? Other feedback on this?

**Scenario 3: research team investment in trial and altruism**

***target POs: Research team investment [7], benefit for future infants [8]***

***Research team member instructions***

Prior work has shown that many parents respond well to learning certain things as they consider research participation. This scenario will focus on how to share information about the **research team’s investment in the trial** and how to **support altruism**. In this scenario, we’ll use a different study as an example to practice these skills, but the approach will be similar to how you would share this information in DIVI.

For this scenario, you’ll play the role of a researcher talking to a [mom][dad] named [Natalie][Chris] about whether [s]he wants [her][his] baby, Olivia, to participate in a study.

*Brief patient history:*

Olivia is a former 23-week infant who is now 7 weeks old. From the chart you see that she was extubated last week after a course that included surgery for NEC. She is currently convalescing on CPAP and her bowel is in discontinuity with an ostomy and mucus fistula.

*Brief research study overview:*

The WEAN-ME study is recruiting extremely preterm babies at 28-32 weeks post-menstrual age to compare standard weaning practices around non-invasive respiratory support with a protocolized approach to weaning based on specific clinical assessment and laboratory values. The research team has clinical and research experience with non-invasive ventilatory support and is hopeful that their new approach will both decrease rates of BPD, decrease long-term respiratory morbidities, and decrease hospital length of stay.

Participation in WEAN-ME includes randomization to the protocolized vs. standard approach to weaning respiratory support. After NICU discharge, participants are followed with in-person assessments and parent surveys at research visits through age 2 years. These are scheduled in conjunction with routine clinical follow-up visits. The team has travel vouchers available for these visits. The team is hopeful that with additional study funding, infant follow up may extend until 5 years of age.

Imagine you’ve already introduced yourself to [Natalie][Chris].

From Olivia’s nurse, you’ve learned [Natalie][Chris] is a single [mom][dad] who has been struggling financially and is worried about all the medical bills from their NICU stay. [S]he is open to discussing research but doesn’t know much about research.

For this scenario, we ask you to focus on the **research team’s investment in trial** and the **benefit for future infants**. Start the scenario by talking a bit about the clinical trial.

***Standardized parent script***

[Natalie][Chris] is a single [mom][dad]. The pregnancy was uneventful until the baby, Olivia, was suddenly born at 23 weeks, had a complicated course complicated by necrotizing enterocolitis, a potentially life-threatening emergency that can have long-term feeding implications, requiring surgery at 3 weeks and will require a second surgery within the 1-2 months to put her intestines back together. She was on the ventilator for the first 6 weeks of life and had multiple unsuccessful attempts off the ventilator, each of which required putting the breathing tube back in, a procedure that felt barbaric and highly stressful to you. Last week she was successfully extubated and seems to be doing much better.

For Natalie: Her ex, Chris, has not been involved throughout the pregnancy or after Olivia’s birth. Natalie has sole custody of Olivia.

For Chris: After his ex, Natalie, gave birth, she decided she could not care for a baby and now Chris has sole custody of Olivia.

As things have stabilized clinically, [Natalie][Chris] has been increasingly worried about paying for the hospitalization and about being able to bring Olivia home. [Natalie][Chris] was expecting to have nearly 4 more months of work before the baby arrived, and [s]he is worried about how much the NICU stay will cost. It has been exhausting to go back and forth to [her][his] apartment (70 min trip on public transit each way) to make sure everything is okay there. While Olivia has been in the hospital, [Natalie][Chris] has started wishing [s]he could do something to keep this from happening to other families. Helping others is how [s]he’s always coped with difficult things that happen. At the same time, [s]he is skeptical about all the various people coming in to talk to [her][him] about things that don’t seem related to Olivia’s care, and [s]he’s had many bad experiences in the medical system over the years, for example being denied care because [s]he didn’t have the right insurance and being looked down on by doctors for how [s]he looked. [S]he’s not sure what to expect from this new doctor coming in to talk about research or what research would mean for [her][him] and [her][his] baby.

[Natalie][Chris] starts off quiet and a bit standoffish. [S]he responds to generic statements about the research and its goals with statements like “Okay” or “I guess” to get them to wrap up and move on. [S]he might ask how much it would cost to be in the research. If the researcher illustrates how the research could benefit other babies, [Natalie][Chris] might share more about wanting to make the system better for other people, saying things like “Doctors don’t really listen to me, but if I can make it better for other parents like me and their babies, I want to do that.” If they probe about [her][his] past experiences using active listening statements and showing empathy, [s]he will gradually open up about [her][his] experiences and begin to engage more with the researcher.

***Facilitated group discussion***

How did the conversation feel? How did it feel to try to bring in these two items? Which things felt easy to talk about and which felt forced? How did [Natalie][Chris] respond? What are some other ways we could bring up these items?

**Scenario 4: options for participation, ongoing connection with family**

***target POs: options for participation [9]; ongoing connection with family [10]***

***Research team member instructions***

Prior work has shown that many parents want help considering reasons for or against participation in a clinical trial and that sharing these reasons can provide a sense that their decision will be supported and respected either way. Families also have shared a desire to be able to easily contact the research team if needed.

This final scenario will draw on skills around supporting decision-making. This scenario will build on our earlier conversation with [Natalie][Chris], single [mom][dad] of Olivia.

For this scenario, please focus on options for participation and support of ongoing connection with the family.

***Standardized parent script***

*See* ***scenario 3*** *above for background about [Natalie][Chris].*

In this scenario, if the researcher identifies helping others as a reason some people participate in research and/or that the time burden may be a reason people don’t participate, [Natalie][Chris] might share that [s]he loves the idea of Olivia being able to help future premature babies and their families but that [s]he is hesitant to commit to something that will mean extra research follow-up visits. [S]he might ask about whether transportation to and from clinic follow-up appointments can be provided. If the researchers explicitly express that families think about these questions differently and asks what is important to [her][him], [s]he might say, “I really think it’s important to help make things better for families in the future… I want to do it but just need to figure out how to make it work for our family.”

If the researcher proactively shares their contact information, [Natalie][Chris] might share [her][his] relief at getting the contact information and being invited to reach out again, maybe saying “I’m sure I’ll have questions later that I’m not thinking about now.” If they explicitly mention following up about something [s]he mentioned before, [s]he might smile and warmly tell them how much [s]he appreciates them taking the time to listen, and that it sounds like a really good study that [s]he’d like to help with.

***Facilitated group discussion***

How did the conversation feel? How did it feel to try to bring in these items? Which things felt easy to talk about and which felt forced? How did [Natalie][Chris] respond? What are some other ways we could bring up these items?
