# Supplementary material for: Development of the Better Research Interactions for Every Family (BRIEF) intervention to support recruitment for neonatal clinical trials: an intervention mapping guided approach
Source: Trials. 2024 Sep 12;25:610. doi: 10.1186/s13063-024-08446-6 (PMC11395641; doi:10.1186/s13063-024-08446-6)
Supplement: Supplementary file 2 — Supplementary Material 2: Fig. S2. BRIEF Assessment Tool. [file 13063_2024_8446_MOESM2_ESM.docx]

Figure 4. BRIEF Assessment Tool [online Supplement]

Please respond to these questions regarding the specific recruitment conversation you just completed for the DIVI study. For each item, please choose which of levels 1-5 (including half levels) best matches your perceptions of that recruitment discussion. These levels are designed so that experienced research team members may select any level for a particular question (an expert may still select 1 or 2 for some questions about some interactions). If you are not sure which level fits best, please review the descriptions and examples and make your best guess.

| **1. Partnership with Clinical Team**  Intent: Research team member demonstrates to the family that the study team is partnered with the patient’s clinical team to show link between research and clinical care. | | | | | |
| --- | --- | --- | --- | --- | --- |
| 1 | | 2 | 3 | 4 | 5 |
| Does not have opportunity to connect with clinical team or discuss this with family | | Shares that clinical team is aware of research team’s approaching the family | Connects with clinical team and shares that they support the research team in approaching the family | Illustrates connection between the clinical and research teams by naming who they connected with before approach | Illuminates a strong connection between the clinical and research teams by naming provider and their belief in patient eligibility |
|  | **Examples** | | | | |
| 1 | Unable to connect with providers about the research study before approaching family. | | | | |
| 2 | “*I connected with your doctor about your potential eligibility for our study.”* | | | | |
| 3 | *“Your doctor said it was alright if I come talk with you about the study.”* | | | | |
| 4 | *“Dr. Smith said it was OK if I come talk with you about the study.”* | | | | |
| 5 | *“Early today I talked with Dr. Smith, who agreed that your baby might be a good candidate for this study”* | | | | |

| **2. Partnership with Bedside Nursing**  Intent: Research team member builds on relationship between family and bedside nursing to support introductory relationship-building between family and the research team. | | | | | |
| --- | --- | --- | --- | --- | --- |
| 1 | | 2 | 3 | 4 | 5 |
| Does not have opportunity to connect with nursing or discuss this with family | | Describes nursing support for research team presence at bedside | Connects with nursing before approach and tells family they have done so | Illustrates partnership with the bedside nurse by sharing that they support the research team approaching the family | Builds on the connection between the family and nursing, by naming the nurse and sharing with parents information gained from connecting with them |
|  | **Examples** | | | | |
| 1 | Unable to connect with bedside nursing about the research study before approaching family. | | | | |
| 2 | *“Nursing knows that research teams like ours often approach families in the NICU.”* | | | | |
| 3 | *“I let your nurse know we’d be chatting about a research study.”* | | | | |
| 4 | *“I let your nurse know we’d be chatting about a research study, and they said this would be a good time.”* | | | | |
| 5 | *“Your nurse Jenny said that now might be an ok time to talk about a potential research study. She mentioned that you guys prefer to keep it quiet in Johnny’s room so I will talk quietly.”* | | | | |
| **3. Family Names**  Intent: Research team member initiates relationship with infant and caregivers by learning how they would like to be addressed and addressing them accordingly. | | | | | |
| 1 | | 2 | 3 | 4 | 5 |
| Proceeds without asking names/roles or omits names during consent conversation | | Primarily uses generic titles or roles (ex: “mom”) in conversation | Variably recalls and uses individuals’ preferred terms | Requests and consistently uses individuals’ preferred terms, pronunciations, and pronouns throughout | Plans ahead by obtaining names, then confirms and uses preferred terms, pronunciations, and pronouns |
|  | **Examples** | | | | |
| 1 | *“Hi, I’m here to talk to you about your baby being in a clinical trial.”* | | | | |
| 2 | *“Are you mom? Great! It’s nice to meet you. Since you are a mom of a baby in the NICU we would like to talk with you about your baby being in a clinical trial.”* | | | | |
| 3 | “*What is your name? It is so nice to meet you, Karen. Since you are a mom of a baby in the NICU, we would like to talk with you about your baby being in a clinical trial.”* | | | | |
| 4 | “*What is your name? It is so nice to meet you, Karen. And who is this? (Referring to baby). As Johnny’s mom, we would like to talk with you about Johnny possibly being in a clinical trial on babies in the NICU.”* | | | | |
| 5 | *“Hello, you must be Johnny’s mom? Is it alright if I call you Karen, or would you prefer Ms. Thompson, or something else? Great! So, Karen, I am here today to talk with you about Johnny possibly being in a clinical trial.”* | | | | |

| **4. Options for Discussing Research**  Intent: Research team member provides families choices to make the discussion of research most productive and comfortable for them. | | | | | |
| --- | --- | --- | --- | --- | --- |
| 1 | | 2 | 3 | 4 | 5 |
| Confirms with family it is an appropriate time to discuss research | | Offers specific options of other times and/or locations to discuss research | Acknowledges that the family may not feel ready to discuss research and offers option to talk later | Empathizes how it may be difficult to discuss research in the NICU and partners with the family to make the experience more comfortable | Individualizes discussion of options by anticipating family’s needs, seeking preferences, and following through to meet preferences |
|  | **Examples** | | | | |
| 1 | *“Is now a good time to hear about a research study?”* | | | | |
| 2 | *“If you want, I can reserve a quiet room for us to chat, and/or I can come back tomorrow if now isn’t a good time*”. | | | | |
| 3 | *“I know you’re going through a lot, so how do you feel about chatting with me about research? If now isn’t the best time, that’s okay, we can also chat tomorrow.”* | | | | |
| 4 | *“It can be hard to talk about research while in the NICU. If now isn’t the right time or place, I could come back later and/or we could find another space to chat. What feels right to you?”* | | | | |
| 5 | *“I’d like to figure out how to make you feel most comfortable in our conversation. From what you’ve told me about your day so far, maybe it would be good for me to give you some space and come back later?”* | | | | |

| **5. Empathy with the NICU Family Experience**  Intent: Research team member acknowledges that the NICU is a challenging place for families and support relationship-building through empathizing with family experiences. | | | | | |
| --- | --- | --- | --- | --- | --- |
| 1 | | 2 | 3 | 4 | 5 |
| Opportunity to connect with family regarding their NICU experience does not arise or is not recognized | | Responds to specific challenges shared by the family | States recognition of the challenges in the NICU | Invites the family to share their NICU experience with open ended questions and responds with empathetic statements | Anticipates and explores family NICU experience, recognizes and responds to subtle emotional cues, and provides empathic support |
|  | **Examples** | | | | |
| 1 | Doesn’t recognize an opportunity to connect emotionally with family. | | | | |
| 2 | Responds to family sharing that the patient has had a recent infection scare with: *“That sounds so hard.”* | | | | |
| 3 | *“I know things can be very challenging for babies and families in the NICU.”* | | | | |
| 4 | *“How are you all doing today?... It sounds like it was a long night, I can’t imagine how stressful that was for your family.”* | | | | |
| 5 | *“I have heard from families about how hard their time in the NICU is. How are you all doing? … Driving hours every day to visit your baby sounds exhausting.”* | | | | |

| **6. Family Needs**  Intent: Research team member supports relationship building by identifying, exploring, acknowledging, and addressing family’s needs. | | | | | |
| --- | --- | --- | --- | --- | --- |
| 1 | | 2 | 3 | 4 | 5 |
| Opportunity to discuss family needs does not arise or is not recognized | | Describes existence of resources for families to get needs or questions addressed | Elicits and acknowledges needs; offers to take action to support getting needs addressed | Explores any unmet needs including responding to parental verbal and non-verbal cues; and acts within role to address as appropriate | Anticipates and identifies needs by preparing to meet the family and directly asking parents their needs; ensures the needs get addressed by the team as a whole |
|  | **Examples** | | | | |
| 1 | No family needs arise or are identified. | | | | |
| 2 | *“The hospital has lots of resources available to help with financial concerns.*” | | | | |
| 3 | *“It sounds like you have questions about next steps for your baby. I can let your doctor know you’d like to talk about that.”* | | | | |
| 4 | *“Can I get you anything before we talk more about the study? … Let me grab you some water, and I will touch base with your nurse to make sure she knows you would like to talk about the new medication schedule.”* | | | | |
| 5 | *“Would you be interested in a resource flyer that I have thatoutlines some of the most used and common family resources, including transportation services? Also, would it be okay if I let our social worker Sam know you’d like a visit to discuss options?”* | | | | |

| **7. Research Team’s Investment in Trial**  Intent: Research team member effectively communicates why this research team is invested in this clinical trial. | | | | | |
| --- | --- | --- | --- | --- | --- |
| 1 | | 2 | 3 | 4 | 5 |
| Describes the overall goals of this trial | | Discusses importance of this trial for the NICU community | Illustrates specific examples of why this trial is valuable to the NICU community | Shares personal reasons why research team members are motivated to do this trial | Formulates a compelling message on how this trial fits within the larger goals of the research team |
|  | **Examples** | | | | |
| 1 | “*This research aims to determine if iron may impact babies’ brain development.”* | | | | |
| 2 | *“Iron is important for brain development and this research study should teach us how to help more babies in the NICU.”* | | | | |
| 3 | *“The team doing this study are experts in iron metabolism and we are all really hopeful that this proves to be helpful to babies in the NICU.”* | | | | |
| 4 | *“The research team was inspired to address iron as it’s so important to the healthy development of babies, like Johnny. Our team is hopeful that this trial will give us another tool to help babies in the NICU thrive.”* | | | | |
| 5 | *“This study is led by a leader in nutritional support for babies like Johnny who are struggling with the challenges of prematurity. The team and I are hopeful that this project will contribute to our mission of helping babies grow in the NICU.”* | | | | |

| **8. Benefit for Future Infants**  Intent: Research team member shares the rationale for the study including jargon-free explanations of why this issue is important to infants and families. Use this explanation to connect the family’s hopes for their own infant with their hopes for other infants in their situation. | | | | | |
| --- | --- | --- | --- | --- | --- |
| 1 | | 2 | 3 | 4 | 5 |
| States that research can help future infants | | States potential benefits of this trial for future infants | Illustrates the connection between research goals and family goals of helping future infants | Proposes a shared goal between family and research team to help infants in the future | Formulates a compelling message of this study’s potential impact on NICU research and connects the message to the individual infant and family |
|  | **Examples** | | | | |
| 1 | *“Research can help future NICU babies.”* | | | | |
| 2 | *“This trial will teach us about how to manage iron supplementation for future babies in the NICU.”* | | | | |
| 3 | *“We are hopeful to learn from families like yours how to better manage iron supplementation for babies in the NICU.”* | | | | |
| 4 | *“We are hopeful that this trial will help Johnny and other babies like Johnny in the future have the best development they can.”* | | | | |
| 5 | *“We are hopeful that this trial will enable NICU babies like Johnny to thrive. For some families, participation is a way of giving hope to other families in the same situation."* | | | | |
| **9. Options for Participation**  Intent: Research team member demonstrates that the decision whether to participate is the family’s choice and that families make different choices. | | | | | |
| 1 | | 2 | 3 | 4 | 5 |
| Lists the potential risks and benefits to participating in the study and offers option to participate | | Describes potential risks and benefits to participation and ensures family understands it’s an individual choice | Explains that different families might think about the potential risks and benefits differently | Shares potential risks and benefits and compares how different families might apply that information to their decision | Individualizes discussion by eliciting family’s values and experience to support their decision to participate or not participate |
|  | **Examples** | | | | |
| 1 | Lists risks and benefits, then tells family they can choose whether to participate or not | | | | |
| 2 | *“I just shared a lot of things to think about. As you’re making your decision, you can think about which of these things are most important to your family.”* | | | | |
| 3 | *“Families weigh these risks and benefits differently. Some families see the risks of the study as outweighing the benefits, and others see the benefits as outweighing the risks.”* | | | | |
| 4 | *“For some families, the time required to do the research activities may feel overwhelming. For others, it may be seen as a way to get more information about their baby’s development.”* | | | | |
| 5 | *“Each family makes the best decision for them about joining research studies, and they also have different approaches to making that decision. After hearing about the study, what stands out as important for your family?”* | | | | |

| **10. Ongoing Connection with Family**  Intent: Research team member provides the information families need to make it easy and comfortable for them to follow up with the research team. | | | | | |
| --- | --- | --- | --- | --- | --- |
| 1 | | 2 | 3 | 4 | 5 |
| Points out research team contact information on study documents and states they can use it to contact the team | | Encourages family to contact research team with any questions using the contact information provided | Acknowledges that families often have questions after the research discussion and expresses research team’s receptivity | Illustrates an example of questions that families may have and highlights how research team can help answer questions | Identifies potential areas of concern based on family’s expressed values and ensures family knows how research team can help |
|  | **Examples** | | | | |
| 1 | *“Our study team’s information is listed at the top of the study consent form if you have any questions or concerns, you can contact us.”* | | | | |
| 2 | *“Our information is at the top of the study consent form. Please reach out to us with any questions. That’s what we’re here for.”* | | | | |
| 3 | *“Families often have questions about the research that come up later. Our contact information is here and we encourage you to ask us any questions you have.”* | | | | |
| 4 | *“Sometimes questions come up later. For example, families will wonder about the timeline for the study activities. Our team can answer all of those questions for* you.” | | | | |
| 5 | *“You mentioned you had questions about our access to your baby’s information. If you want more detailed information, I can ask the study doctor to come talk to you now, or if things come up later you can reach us at the number listed here.”* | | | | |
